# Development of the physical education knowledge questionnaire-Spain (PEKQ-S): feasibility, validity and reliability for Spanish children aged 6–12 years

**DOI:** 10.3389/fspor.2025.1737905

**Published:** 2026-01-23

**Authors:** Raquel Pastor-Cisneros, María Mendoza-Muñoz, José Francisco López-Gil, Jorge Carlos-Vivas

**Affiliations:** 1 Physical Activity for Education, Performance and Health (PAEPH) Research Group, Faculty of Sport Sciences, University of Extremadura, Cáceres, Spain; 2 Department of Communication and Education, Universidad Loyola Andalucía, Sevilla, Spain; 3 School of Medicine, Universidad Espíritu Santo, Samborondón, Ecuador; 4 Vicerrectoría de Investigación y Postgrado, Universidad de Los Lagos, Osorno, Chile

**Keywords:** assessment, physical activity, physical literacy, SPLA-C, validation

## Abstract

**Purpose:**

Physical literacy (PL) encompasses the motivation, confidence, physical competence, and knowledge necessary for engaging in physical activity throughout one's life. Although tools such as the Canadian Assessment Physical Literacy (CAPL-2) assess PL globally, they lack cultural alignment with the Spanish context in terms of their cognitive components. This emphasizes the importance of developing a context-specific tool for assessing PL-related knowledge in Spanish primary education. This study aims to develop and validate the Physical Education Knowledge Questionnaire-Spain (PEKQ-S) for children aged 6–12, aligning it with the national physical education (PE) curriculum and evaluating its factor structure and reliability.

**Methods:**

The PEKQ-S was developed through curriculum review, expert consultation, and student input via open-ended and closed-ended questions. Its feasibility and reliability were tested in 145 Spanish primary school children aged 6–12, and its structure was evaluated via exploratory factor analysis.

**Results:**

Exploratory factor analysis identified two correlated factors across nine items. The PEKQ-S showed acceptable internal consistency [Cronbach's alpha (*α*) = 0.508–0.832] and fair to substantial test–retest reliability [intra-class correlation coefficient (ICC) = 0.337–0.696]. Moderate correlations were found between factors (r = 0.362), and most items demonstrated good reproducibility and stability.

**Conclusion:**

The PEKQ-S is a valid, reliable, and feasible tool for assessing PE-related knowledge linked to the PL in Spanish children aged 6–12. It represents the first instrument of its kind in Spain and forms part of the Spanish Physical Literacy Assessment for Children (SPLA-C) model.

## 1 Introduction

Physical literacy (PL) is defined by the Bulletin of the International Council of Sport Science and Physical Education of the United Nations Educational, Scientific and Cultural Organization (UNESCO) as the motivation, confidence, physical competence, knowledge and understanding to value and participate in a physically active lifestyle (1).

According to Whitehead (2), the term “knowledge” encompasses the knowledge and understanding component of PL, including movement (how to move), performance (evaluating movement) and health and fitness (assessing exercise, its benefits, relaxation techniques, etc.). Furthermore, in Cutter's recent study (3), experts determined that content knowledge, creativity, decision-making, and safety and risk, among other factors, were considered part of the list of cognitive elements crucial for assessing the cognitive domain.

Currently, there are different recognized assessments of PL (4–8), but the most widely used assessment worldwide is the Canadian Assessment of Physical Literacy (CAPL-2) (6), which has been validated and applied in numerous countries (9–12). Within the CAPL, there are standardized protocols to assess each of the components: motivation and confidence (13, 14), physical competence (15–17), engagement in daily physical activity (18) and knowledge and understanding of physical activity (19).

The CAPL-2 has been applied to assess PL in Spanish children and adolescents (20–22). However, the main problem derived from the application of this tool to assess PL in our population context was mainly sociocultural (23) and educational-curricular differences with respect to the Canadian population. The cognitive domain was the most penalized with respect to these differences, in which no significant results were found in the PL studies carried out in Spain (21, 22, 24). Therefore, even though knowledge of the contents encompassing PA is part of the national physical education (PE) curriculum in Spain, no standardized measure has been identified to assess the knowledge and understanding of PL. In this context, creating a questionnaire adapted to the context and needs of our population has become a priority line of research for assessing PL in our country.

Edwards et al. (25) stated that PE is considered to be the most popular context in which to associate PL. Although PE should be abstracted from the environment in which it occurs (26, 27), the authors pointed out that it is appropriate to apply this concept in schools. In this way, the whole field of education, especially PE, would be enriched, and the evaluation and monitoring of PL would be more accessible to the younger population. Therefore, to create this synergy between PL and PE, it is necessary to have a knowledge questionnaire that focuses on assessing the contents of the national PE curriculum that are related to the concept of PL.

Owing to the importance of acquiring PL at the earliest ages and the lack of available instruments for the assessment of the cognitive domain, this study aims to develop and validate a standardized assessment of the knowledge of the national PE curriculum related to PL for primary school children (6–12 years old), resulting in the Physical Education Knowledge Questionnaire in Spain (PEKQ-S). It is also intended to evaluate its factor structure and reliability.

## 2 Material and methods

### 2.1 Study design

**Figure 1:**
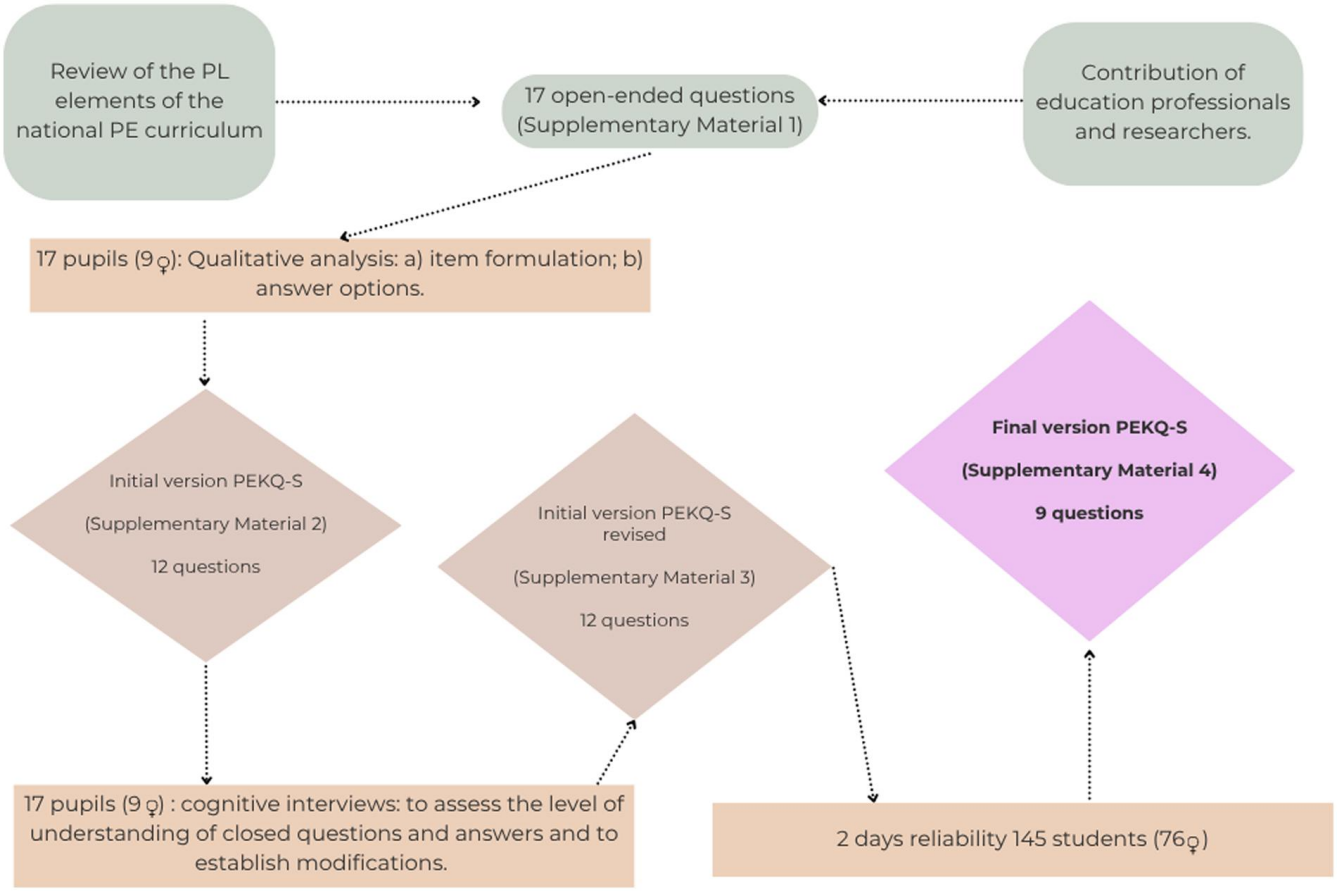
Overview of research to develop the PEKQ-S.

The design and development of the PEKQ-S was carried out through a series of procedures, as described in Figure 1. Initially, the proposed content was identified through a review of the elements of the national PE curriculum that relate to PL, together with input from educational practitioners and researchers in the field of PL. After several rounds of discussion and consensus, a file of potential open-ended questions (Supplementary Material 1) was created.

The answers were subsequently analyzed in a qualitative way, thus generating the items and the list of response options in a closed-ended format. In addition, this initial closed-ended question-and-answer format (Supplementary Material 2) was subjected to a cognitive interview with 10 schoolchildren (5 boys and 5 girls).

The feasibility of the initial PEKQ-S (Supplementary Material 3) was assessed by having schoolchildren (aged 6–12) answer the closed-ended questions in pencil and paper formats. Response errors and individual student errors were assessed. The reliability of the PEKQ-S was assessed on two separate occasions, with a 15-day interval between them. After the reliability of the PEKQ-S was analyzed, the final version of the 9-question questionnaire was obtained (Supplementary Material 4).

### 2.2 Sample size calculation

The required sample size for this study was estimated *a priori* on the basis of test‒retest reliability analysis to evaluate the stability of the scores over time. Specifically, to estimate the instrument's temporal reliability, the intraclass correlation coefficient (ICC) is calculated on the basis of two measurements taken 15 days apart. Thus, the required sample size for this analysis was calculated via G*Power 3.1.9.4 (28) with the repeated-measures ANOVA model (within-subjects design) selected. Assuming a medium effect size (f = 0.25), a significance level of *α* = 0.05, a statistical power of 1-*β* = 0.95 and an expected correlation between the two sets of measurements of 0.5, the required sample size was 54 participants.

### 2.3 Participants

A convenience sample of 145 schoolchildren aged between 6 and 12 years from five schools in the Autonomous Community of Spain, whose parents gave their written consent, participated in the study.

### 2.4 PEKQ-S procedure and development

The content areas for the PEKQ-S were systematically identified through an exhaustive review of the national PE curriculum (29), which was valid for all the Autonomous Communities of Spain. Table 1 lists the key elements of the national PE curriculum common to all grades of primary school: competences, basic knowledge and specific aspects related to PL. The content of these elements common to all primary grades was analyzed: (1) Healthy lifestyle, knowing what it is to be healthy (including eating habits and body hygiene); (2) Benefits of practising PA; (3) 24-hour patterns (PA, sedentary time, sleep); (4) Safety before, during and after PA; (5) Recognition and own choice of PA; (6) Motor skills; (7) Physical abilities; (8) Teamwork; (9) Emotional self-regulation in sport and physical-sports activities; (10) Body expression; (11) Active play; (12) Active transport; (13) Possibility of doing sport in any environment (water, land, air); and (14) Physical-sports activities in the natural environment. This was followed by proposals for open-ended questions on key aspects related to PL (Supplementary Material 1). Feedback on the clarity and wording of the questions was obtained from several primary PE teachers. The children's responses to the open-ended questions were used to identify closed-ended response options to be included in the initial PEKQ-S (Supplementary Material 2). Afterwards, a cognitive interview was conducted with 17 children on their understanding of the closed-ended questions and responses, resulting in the initial revised version of the PEKQ-S (Supplementary Material 3).

**Table 1:** Key elements of the Spanish PE curriculum and related open-ended questions.

Key elements of the Spanish PE curriculum			Open-ended questions
Competences	Basic knowledge	Specific key issues to be assessed	
CE1. Adopt an active and healthy lifestyle, regularly practising physical, recreational and sporting activities, adopting behaviors that promote physical, mental and social health, as well as measures of individual and collective responsibility during motor practice, in order to internalize and integrate systematic physical activity habits that contribute to well-being.	A. Active and healthy life	1. Healthy lifestyle, knowing what it means to be healthy (including eating habits and body hygiene).	1.1. Do you think it is important to be physically fit? Yes/No Why? 1.2. Draw a line to all the words that you think describe “what it is to be healthy”. (Sample answers: Disease-free, look good, feel good, be flexible, be happy, be popular, have stamina, have strong muscles, be attractive, be slim, good nutrition, exercise). 1.3. Please circle the healthy foods. Put an X on the foods that are not good for you. 1.4. What do you think it means to be physically fit? What does a fit and an unfit person do? 1.5. What personal hygiene habits do you think are most important after sport/What things do you do to keep yourself clean after sport?
		2. Benefits of practising PA.	2.1. Name 3 things you like about playing sport or being physically active. 2.2. Name 3 things you DON'T like about being physically active or playing sport
	B. Organization and management of the PA.	3. 24-hour patterns (PA, sedentary time, sleep)	3.1. Name 2 activities that you think are healthy to do after school. (Possible answers: Playing video games, reading, doing homework, playing outside with friends, practicing with my sports team, taking the dog out, chatting, watching TV). 3.2. Name 2 activities that you think are NOT healthy to do after school. (Possible answers: Playing video games, reading, doing homework, playing outside with friends, practicing with my sports team, taking the dog out, chatting, watching TV.) 3.3. How much time do you watch TV after school/after dinner/on weekends? a. Less than one hour b. 1–2 c. 3–4 d. More than 5 e. None 3.4. How much time do you spend on the computer after school/after dinner/on weekends? a. Less than one hour b. 1–2 c. 3–4 d. More than 5 e. None 3.5. How much time should you and other Canadian children spend being physically active each day? __minutes
		4. Safety before, during and after PA.	4.1. What do you do to prepare yourself before doing sport? List as many examples as you can think of. 4.2. What do you do after exercise to take care of your body and recover? 4.3. What do you do to feel safe before you exercise and while you are exercising?
		5. Recognition and own choice of physical activities.	5.1. When you are not at school, what sports or activities do you do in the summer/spring? 5.2. When you are not at school, what sports or activities do you do in autumn/winter? 5.3. What kind of physical activities do you like to do most and why? 5.4. What steps do you need to take to improve in a sport or physical activity that you like?
CE2. Adapt the elements of the body scheme, physical, perceptual-motor and coordination capacities, as well as motor skills and abilities, applying processes of perception, decision and execution appropriate to the internal logic and objectives of different situations, in order to respond to the demands of motor projects and motor practices with different purposes in daily life contexts.	C. Problem solving in motor tasks	6. Motor skills.	6.1. Running, throwing, jumping, catching and kicking are skills that we use all the time in physical activities. Why do you think these are important? 6.2. All the athletes in the pictures are performing the same action. What sporting skill are they doing? (Several athletes throwing)
		7. Physical capabilities.	7.1. Cardiorespiratory fitness is: (a) The ability of the muscle to contract. b) The ability of the heart to pump blood and the ability of the lungs to supply oxygen c) Our heart rate d) Our ability to run and do the sports we like to do. 7.2. Fill in the gaps with the words. Story to be completed with words: cardiorespiratory, pulse, well, fun, strength, endurance, lungs, 90 and heart. 7.3. What exercises do you like best to improve your endurance and why? 7.4. What activities help you feel stronger? 7.5. What do you think you need to run faster or jump higher?
CE3. Develop processes of self-regulation and interaction within the framework of motor practice, with an empathetic and inclusive attitude, making use of social skills and attitudes of cooperation, respect, teamwork and sportsmanship, regardless of the ethno-cultural, social, gender and ability differences of the participants, in order to contribute to coexistence and ethical commitment in the different spaces in which they participate.	D. Emotional self-regulation and social interaction in motor situations	8. Teamwork.	8.1. Give 2 examples of how you work as a team with your friends during a game/sport. 8.2. How do you resolve conflicts that may arise during a physical activity with your friends or classmates? 8.3. What rules do you follow when playing with your friends to make sure that everyone has fun?
		9. Emotional self-regulation in sport and physical-sports activities.	9.1. How do you motivate yourself if you find a physical activity difficult? 9.2. How do you feel when you win or lose in a game/sport? What do you do to manage those feelings?
CE4. Recognize and practice different recreational, physical-sports and artistic-expressive manifestations of motor culture, valuing their influence and their aesthetic and creative contributions to traditional and contemporary culture, in order to integrate them into the motor situations regularly used in everyday life.	E. Manifestations of motor culture	10. Corporal expression.	10.1. How can dance or artistic movement be used to express emotions and tell stories? Do you know of any examples? 10.2. How do you collaborate with your peers to create a choreography as a group? What challenges and benefits do you find in the process? 10.3. Describe a time when you created a movement composition that you were proud of. 10.4. List, draw or describe movements that can help you to express your emotions and thoughts. 10.5. What do you understand by body language? 10.6. Give an example of how you can use your body to show an emotion without speaking. 10.7. What movements or gestures do you use to express joy or sadness? 10.8. How do you think music can help you move in different ways?
		11. Active play	11.1. What active games do you like to play at recess and why? 11.2. Can you describe an active game you played recently and what you liked about it? 11.3. Can you tell me about a traditional game you know and how it is played?
CE5. Value different natural and urban environments as contexts for motor practice, interacting with them and understanding the importance of their conservation from a sustainable approach, adopting measures of individual responsibility during the practice of games and physical-sports activities, in order to carry out an efficient and respectful practice with the environment and participate in its care and improvement.	F. Efficient and sustainable interaction with the environment	12. Active transport.	12.1. Please circle how you get to school most of the time in spring/summer (good weather) Bus, car, walk, skate, skateboard, scooter, bike..How long does it take you to get there? 12.2. Please circle how you get to school most of the time in winter/autumn (cold or snowy weather) Bus, car, walk, skate, skateboard, scooter, bike..How long does it take you to get there? 12.3. Can you tell me about a time when you decided to walk or cycle to get somewhere, instead of going by bus or car? How did you feel? 12.4. Why do you think it is important to walk or cycle instead of using the car? 12.5. What are the benefits of active transport (walking, cycling, skateboarding) for you and the planet?
		13. Possibility of doing sport in any environment (water, land, air).	13.1. Underline the activities that take place in the aquatic environment, circle those that take place in the terrestrial environment and make a cross in those that take place in the aerial environment (Drawings with scuba diving, sailing, paragliding, rugby, etc.).
		14. Physical-sports activities in the natural environment.	14.1. What activities do you know of that can be done outdoors? 14.2. Can you name 3 things that you enjoyed on an excursion or outing in nature where you were physically active? 14.3. Can you tell me a sport or game that you have played in: a park, forest or beach? 14.4. What materials or equipment do you need to do your favorite outdoor activities? 14.5. Can you invent a physical activity that can be done in nature and explain to me how to play it?

The original information in Spanish is included as supplementary material in Supplementary Table S1*.*

### 2.5 Reliability of the PEKQ-S

Test-retest reliability data were collected from a sample of schoolchildren (*n* = 145) who were asked to complete the PEKQ-S twice, with an interval of 15 days between the two measurements. A 15-day interval was selected to balance the risk of recall bias with the likelihood of genuine changes in knowledge in children. The test‒retest reliability of the PEKQ-S responses was assessed using the intraclass correlation coefficient (ICC).

### 2.6 Exploratory factor analysis (EFA)

The maximum likelihood method was used to identify two factors, each comprising at least three items (30, 31). Bartlett's test of sphericity and the Kaiser–Meyer–Olkin (KMO) measure of sampling adequacy were both used to determine whether the matrix as a whole was appropriate for factor analysis. Bartlett's test of sphericity was significant [Bartlett's test = 190.043, degrees of freedom (df) = 66, *p* < 0.001], and the KMO measure was greater than 0.5 (KMO test = 0.693). This indicates that factorability can be assumed. Once the number of factors had been defined, oblimin with the Kaiser normalization rotation method was applied.

### 2.7 Scoring of the PEKQ-S

The scores for each of the two factors, each question and a total knowledge score are detailed in Table 2 below. The total knowledge scores range from 0 to 90 points, with higher values representing greater mastery of PE content related to PL. Each of the questions is scored between 0 and 10 points. The 9 items include all the basic knowledge contained in the national PE curriculum, except for the basic knowledge “D. Emotional self-regulation and social interaction in motor situations”. This exception is because this element works intrinsically through the questionnaires included in the “Motivation and Confidence” component of the complete Spanish Physical Literacy Assessment for children (SPLA-C) (32) model.

**Table 2:** Labeling and wording of the items of the final version of the PEKQ-S. scoring and coding.

Factor	Basic knowledge (RD 157/2022)	Item	Scoring and coding
Active lifestyles and safe management of physical activity	A. Active and healthy life	1. Do you think it is important to do sport or physical activity on a regular basis? Please tick the correct answer. a) Yes, it is important because physical activity/sport is good for your health. b) Yes, it is important to have a very low weight. c) No, it is not important. It is just fun. d) No, it is not important. In fact, doing sport is dangerous for your health.	Coding: the correct answer has a value of 1. Incorrect answers, blank answers or several marked answers correspond to a value of 0. The correct answer is (a). Correctly answering this question will earn you 10 points out of a possible total of 90.
	A. Active and healthy life	2. Think about which expressions or phrases describe “what it is to be healthy”. Eating well/Doing sport/Not moving/Sleeping 4 h/Not having enough muscle strength/Being clean and tidy/Playing with the mobile phone in bed/Eating sweets/Being fit/Spending too many hours on the sofa.	Coding: each marked answer is worth 1. Each blank answer is worth 0. Correct answers are in bold: **Eating well/Doing sport/**Not moving/Sleeping 4 h/Not having enough muscle strength/**Being clean and tidy**/Playing with the mobile phone in bed/Eating sweets/**Being fit**/Spending too many hours on the sofa. Right combination: 1-1-0-0-0-1-0-0-1-0 Correctly answering this question will earn you 10 points out of a possible total of 90. Each correct answer to this question is worth 2.5 out of a possible 10 points.
	A. Active and healthy life	3. How much time a day do you think children should do physical activity or sport? Mark the correct answer. a) maximum 20 min a day. b) minimum 1 h a day. c) 5 min a day. d) 6 h a day.	Coding: the correct answer has a value of 1. Incorrect answers, blank answers or several marked answers correspond to a value of 0. The correct answer is (b) Correctly answering this question will earn you 10 points out of a possible total of 90.
	B. Organization and management of physical activity	4. Circle the images that you think are necessary to do physical activity or sport in a healthy and safe way.	Coding: each marked answer is worth 1. Each blank answer is worth 0. Correct answers are: (1), (5) y (7). Right combination: 1-0-0-0-1-0-1 Correctly answering this question will earn you 10 points out of a possible total of 90. Each correct answer to this question is worth 3.4 out of a possible maximum10 points.
	B. Organization and management of physical activity	5. Point out or circle which games you think are more active, i.e., you move around while playing.	Coding: each marked answer is worth 1. Each blank answer is worth 0. Correct answers are: (1), (4), (5), (6) y (7). Right combination: 1-0-0-1-1-1-1 Correctly answering this question will earn you 10 points out of a possible total of 90. Each correct answer to this question is worth 2 out of a possible 10 points.
Movement, expression, and interaction with the environment	A. Active and healthy life	6. Not doing physical activity or sport on a regular basis can..Mark the correct answer. a) improve mood. b) help keep the body fit and strong. c) help you make new friends and work with others as a team. d) increase the risk of disease.	Coding: the correct answer has a value of 1. Incorrect answers, blank answers or several marked answers correspond to a value of 0. The correct answer is (d) Correctly answering this question will earn you 10 points out of a possible total of 90.
	C. Problem-solving in motor tasks	7. Write below each picture which basic physical ability is being used: Strength, Speed, Endurance or Flexibility.	Coding: Each blank answer has a value of 0 and each correct answer has a value of 1. If two abilities have been mentioned, the answer is taken as wrong 0. The correct answers are: 1-Fuerza; 2-Velocidad; 3-Flexibilidad; 4-Resistencia. Correctly answering this question will earn you 10 points out of a possible total of 90. Each correct answer to this question is worth 2.5 out of a possible 10 points.
	E. Expressions of motor culture	8. What is body language? Mark the correct answer. a) It is sitting properly. b) It is washing up after sport. c) The way we use our body to communicate something (emotions, thoughts. etc.) without words. d) The way we write with a pencil.	Coding: the correct answer has a value of 1. Incorrect answers, blank answers or several marked answers correspond to a value of 0. The correct answer is (c) Correctly answering this question will earn you 10 points out of a possible total of 90.
	F. Efficient and sustainable interaction with the environment	9. Write below each picture what sport it is and where the sport takes place: water, land or air.	Coding: Each blank answer has a value of 0 and each correct answer has a value of 1. “Medio” and “Deporte” are considered as separate answers for scoring, so one of them can be correct and one of them not. Correctly answering this question will earn you 10 points out of a possible total of 90. Each correct answer to this question is worth 0.85 out of a posible maximum 10 points.

RD, royal decree.

The original information in Spanish is included as supplementary material in Supplementary Table S2.

Bold indicates the correct answers.

### 2.8 Statistical analysis

All the information collected was entered into a database specifically designed for this study. The validation procedure comprised the assessment of construct validity, reliability, and measurement error.

Construct validity was examined using EFA, conducted via the Statistical Package for the Social Sciences (SPSS, version 25.0; IBM SPSS Inc., Armonk, NY, USA). The maximum likelihood method was applied to extract the factors. Given the nature of the data, a polychoric correlation matrix (33) was employed, and the optimal number of factors was determined through parallel analysis (34). Once the number of factors had been identified, Oblimin with the Kaiser normalization method was selected to define factor simplicity and structure. The KMO test and Bartlett test of sphericity were used to assess sampling adequacy (35). Coefficients lower than 0.30 were deleted.

Reliability was assessed through internal consistency and test–retest reliability. A test–retest test was subsequently conducted 15 days apart. The normality and homogeneity of all analyzed variables were checked via the Shapiro–Wilk test. The data are presented as the means and standard deviations (SDs) for both the initial and follow-up tests. The internal consistency and reliability of each question and the total PEKQ-S score were determined via Cronbach's alpha [*α*] coefficient. Cronbach's *α* was interpreted as follows (36): <0.5, unacceptable; ≥0.5 to <0.6, poor; ≥0.6 to <0.7, questionable; ≥0.7 to <0.8, acceptable; ≥0.8 to <0.9, good; and >0.9, excellent. Test–retest reliability or reproducibility was assessed by calculating the intraclass correlation coefficient (ICC) with a 95% confidence interval (CI) (37). A two-way random effects model, single measures, absolute agreement, was used. The ICC values were interpreted according to the criteria proposed by Landis and Koch (38): <0.20, slight agreement; 0.21–0.40, fair; 0.41–0.60, moderate; 0.61–0.80, substantial; and >0.80, almost perfect.

Measurement error (absolute reliability) was quantified using the standard error of measurement (SEM) and minimum detectable change (MDC) (39). Furthermore, Spearman's correlation was employed to examine the relationship between each question and the overall score. The alpha level was set at *p* < 0.05 for all tests.

The study design, validation procedures, and reporting were guided by the Consensus-based Standards for the selection of health Measurement INstruments (COSMIN) guidelines, ensuring adequate assessment of validity, reliability, and measurement error.

## 3 Results

Table 3 shows the structure and factor loading of each item. The factor solution comprises two correlated factors: (1) movement, expression, and interaction with the environment and (2) active lifestyles and safe management of physical activity. Following EFA, questions 5, 8 and 11 were excluded. Therefore, a factor structure consisting of nine items grouped into two factors was extracted. Each factor comprises five items. Factor 1 (movement, expression, and interaction with the environment) comprised questions 6, 9, 10, and 12, whereas factor 2 (active lifestyles and safe management of physical activity) included questions 1, 2, 3, 4, and 7.

**Table 3:** PEKQ-S rotated the factor solution and factor loading.

Item	Factor 1	Factor 2
Q12. Sport and environment	0.585	
Q6. Consequences of not engaging in PA	0.535	
Q10. Body expression	0.533	
Q9. Basic physical capacities	0.404	
Q2. Healthy life		0.570
Q3. Duration of PA		0.484
Q1. Importance of PA		0.432
Q4. Safety in the practice of PA		0.420
Q7. Active game		0.353

Extraction method: maximum likelihood; rotation method: Oblimin with Kaiser normalization.

Table 4 displays the correlations between the PEKQ-S factors: (1) Movement, expression, and interaction with the environment and (2) active lifestyles and safe management of physical activity. Moderate associations were observed between the two factors (rho = 0.362).

**Table 4:** PEKQ-S interfactor correlation matrix.

Factor	Factor 1: movement, expression, and interaction with the environment	Factor 2: active lifestyles and safe management of physical activity
Factor 1: movement, expression, and interaction with the environment	1.000	0.362
Factor 2: active lifestyles and safe management of physical activity	0.362	1.000

Extraction method: maximum likelihood; rotation method: Oblimin with Kaiser normalization.

Table 5 illustrates the internal consistency, reproducibility, and systematic differences of the PEKQ-S. Overall, the internal consistency ranged from poor to good for all questions, factors, and total scores of the questionnaire (Cronbach's *α* ranging from 0.508 to 0.832), except for question 8 (Cronbach's *α* = 0.164). All the questions from the initial and follow-up tests were significantly correlated with the total PEKQ-S score (rho > 0.2).

**Table 5:** Reliability, test‒retest, and systematic differences in the PEKQ-S.

Question	Test (*n* = 145)			Retest (*n* = 145)				Reliability Test					
	M	SD	Question-total correlation	M	SD	Question-total correlation	Cronbach's *α*	ICC (95% CI)	*p* value^†^	SEM	%SEM	MDC	MDC%
Q1	6.78	2.22	.488**	7.24	1.37	.307**	.574	.392 (.245–.521)	.007	1.17	16.7	3.25	46.3
Q2	6.79	1.31	.405**	7.15	0.83	.232**	.574	.383 (.232–.516)	<.001	0.70	10.0	1.94	27.8
Q3	5.59	3.28	.628**	5.84	3.12	.545**	.722	.565 (.443–.666)	.297	1.69	29.5	4.68	81.8
Q4	7.17	0.94	.327**	7.31	0.73	.283**	.508	.337 (.186–.473)	.088	0.59	8.1	1.62	22.4
Q5	7.14	1.61	.234**	6.88	2.07	.180*	.558	.385 (.238–.515)	.132	1.22	17.5	3.39	48.4
Q6	6.10	2.93	.617**	6.10	2.93	.615**	.666	.501 (.368–.614)	>.999	1.69	27.8	4.69	76.9
Q7	6.10	1.25	.231**	6.36	1.00	.224**	.594	.413 (.269–.539)	.012	0.72	11.5	1.99	31.9
Q8	6.72	1.54	.339**	6.80	1.31	.335**	.164	.090 (−.075–.249)	.601	1.30	19.3	3.61	53.4
Q9	6.79	1.82	.431**	6.66	1.82	.421**	.819	.693 (.599–.769)	.275	0.77	11.5	2.15	31.9
Q10	4.50	3.69	.631**	4.45	3.70	.620**	.803	.672 (.572–.752)	.835	1.64	36.6	4.55	101.6
Q11	6.47	2.60	.398**	6.57	2.48	.424**	.430	.275 (.117–.419)	.683	1.92	29.4	5.32	81.5
Q12	6.04	1.39	.542**	6.33	1.08	.471**	.832	.696 (.587–.778)	<.001	0.51	8.2	1.40	22.7
Total PEKQ-S score	76.18	11.88	N/A	77.70	9.98	N/A	.806	.670 (.569–.751)	.041*	6.28	8.2	17.40	22.6

Q, question; M, mean; SD, standard deviation; 95% CI, confidence interval of 95%; ICC, intraclass correlation coefficient; SEM, standard error of measurement; %SEM, standard error of measurement as a percentage; MDC, minimum detectable change; N/A, not applicable.

† Friedman test *p* values. The item-total correlation refers to the magnitude of the association between each item and its domain.

** Significant correlation at *p* < 0.01.

* Significant correlation at *p* < 0.05.

The reproducibility outcomes revealed fair to substantial test–retest reliability for each question and the total PEKQ-S score (ICC = 0.337–0.696), except for question 8, for which slight agreement was reported (ICC = 0.090). The SEM and SEM% values for each question and the total PEKQ-S score ranged from 0.51–6.28 and from 8.1–36.6, respectively. Similarly, the MDC and MDC% values for each question and the total PEKQ-S score ranged from 1.4–17.4 and from 22.4–101.6, respectively.

Finally, comparison outcomes revealed no significant differences for any of the questions or the total PEKQ-S score (*p* > 0.05), except for Q1 (*p* = 0.007), Q2 (*p* < 0.001), Q7 (*p* = 0.012), Q12 (*p* < 0.001) and the total PEKQ-S score (*p* = 0.041).

## 4 Discussion

This study describes the development process and initial findings on the validity of the PEKQ-S as a tool for evaluating the knowledge of the national PE curriculum related to PL among schoolchildren (aged 6–12). The factor structure, internal consistency and interrater reliability of the PEKQ-S were examined. The results revealed a clear bifactor structure, good overall internal consistency, and substantial test‒retest reliability, with a few exceptions regarding individual items.

According to Cutter's study (3), experts indicated that the cognitive domain of PL should primarily encompass knowledge and understanding of the following: the benefits of being active; opportunities and responsibility for PA practice; how to adopt a nonsedentary lifestyle; problem solving; and planning. On this basis, a comprehensive review of the national PE curriculum was conducted, selecting items closely related to PL knowledge for the development process. Longmuir et al. (19) also developed questionnaires to assess PL knowledge, with input from education professionals and PL expert assessors, as in our study. Once the items for the initial version of the PEKQ-S had been established, content validity was improved by conducting cognitive interviews and an external comprehensibility check with schoolchildren and teachers. This methodology has been shown to improve the validity of questionnaire items (40), thereby increasing confidence in the findings of our study.

Exploratory factor analysis of the PEKQ-S revealed a clear bifactor structure, which is consistent with previous studies identifying multiple dimensions of PE knowledge (41, 42). The identified bifactor solution reflects components that are theoretically aligned with PE knowledge, differentiating between “Movement, Expression and Interaction with the Environment” (Factor 1) and “Active Lifestyles and Safe Management of Physical Activity” (Factor 2). The moderate correlation (r = 0.362) between the two factors supports the hypothesis that, while they are interrelated, they represent distinct dimensions of the overall construct. This differentiation between factors related to motor expression and those linked to active lifestyles is consistent with theoretical models that view PE as a multidimensional construct (43, 44). Similarly, PL itself is treated as a multidimensional construct (45). Notably, the curriculum content selected for the PEKQ-S is related to PL, which further reinforces our bifactor structure.

The scale demonstrated good overall internal consistency (Cronbach's *α* = 0.806), which is similar to that reported for other PL assessment instruments, such as the PLAYself (8), the Perceived Physical Literacy Scale for Adolescents (PPLSA) (46) and the PL-C Quest (7). The PL-C Quest (7) is specifically applied to the same target population as in our study. Despite a higher overall internal consistency (Cronbach's *α* = 0.92), the part of the scale referring to the cognitive domain (consisting of seven items) showed lower consistency (Cronbach's *α* = 0.72) than reported in our results. In our study, however, item 8 showed very low consistency (Cronbach's *α* = 0.164), suggesting that it may not adequately measure the same construct as the other items in its factor. However, as in previous studies (47, 48), items with low performance were also observed, indicating the need for revision or reformulation to improve their alignment with the overall construct. In our study, we decided to eliminate item 8, as the basic knowledge covered by it was already included in other PEKQ-S items. Although item 8 was removed due to low internal consistency, the content it addressed is adequately covered by other items within the “Movement, Expression, and Interaction with the Environment” factor. Therefore, its exclusion does not compromise the conceptual representation of this factor or the overall assessment of the cognitive domain of physical literacy.

Like our findings, other instruments containing items assessing the cognitive domain of PL have reported Cronbach's *α* values ranging from moderate to good (0.6–0.8) (49, 50), reflecting acceptable internal consistency for recently developed scales in educational settings. A previous study by Longmuir et al. (19) reported higher reliability than our questionnaire did; however, this may be because the interval between the test and retest was shorter, which may have led to greater concordance in the responses due to recall and experience. In terms of reproducibility, the ICC values obtained (0.337–0.696) are comparable to those reported in previous research on self-reported measures of PL (24, 51). However, our findings diverge for question 8, which exhibited poor consistency and reliability. This highlights the need to eliminate it, as its inclusion compromises the usefulness of the questionnaire in longitudinal assessment contexts. A similar issue was found with other PL tools (49, 52), which require item refinement to improve psychometric performance. Although the overall test–retest reliability of the PEKQ-S was acceptable, several items (Q1, Q2, Q7, Q12) demonstrated moderate ICC values (<0.50) and statistically significant test–retest differences. These findings warrant careful interpretation, particularly in longitudinal applications. Moderate ICCs at the item level are common in self-reported knowledge instruments administered to children and may reflect developmental variability, differences in attention or comprehension, or genuine short-term learning effects rather than poor measurement quality. From a practical perspective, the PEKQ-S is more robust for assessing stability and change at the scale or factor level than for detecting subtle changes in individual items. Item-level ICCs below 0.50 should therefore be interpreted cautiously, whereas changes exceeding the established measurement error thresholds at the total or factor level are more likely to represent meaningful change. Overall, these results suggest that the PEKQ-S is suitable for group-level assessment and for monitoring meaningful improvements in PE-related knowledge over time, while minor item-level fluctuations are expected and should be considered in the context of typical learning processes in school-aged children.

One of the main limitations of our study is the small sample size, which comes from schools in the autonomous community of Extremadura. Therefore, it is unclear whether the study sample is representative of the Spanish child population as a whole. Future studies should confirm the structure of this questionnaire via a larger, more representative sample that includes a greater number of schools and autonomous communities.

With respect to the age range, it should be noted that samples from the first year of primary school were excluded, as the questionnaire was not suitable for this stage of education and could have affected the responses. However, there is a good reason for excluding this group: it is the first year in which PE is taught. Therefore, asking them about content or learning that they have not yet received at school might not be valid. Therefore, if PL assessment instruments are to be developed for early childhood education in future studies, the first year of primary school should also be included alongside preschool.

Finally, it should also be noted that the self-reported nature of the PEKQ-S itself is considered a limiting factor, as it makes the instrument susceptible to recall bias, subjectivity and social desirability.

Given the lack of significant results in the cognitive domain in all previous studies (12, 39) that have applied the CAPL-2 in Spain, this study is the first to offer the development and validation of a knowledge questionnaire adapted to the sociocultural and curricular-educational characteristics of Spanish children in terms of PL. Specifically, the PEKQ-S is the first instrument to assess the knowledge of primary school children in relation to the contents of the national PE curriculum for PL, providing a comprehensive measurement of two differentiated factors: “Movement, Expression and Interaction with the Environment” (Factor 1) and “Active Lifestyles and Safe Management of Physical Activity” (Factor 2). This facilitates more targeted instruction and curriculum planning on the basis of students’ knowledge levels. The questionnaire can identify specific areas of weakness in PE knowledge across different age groups or school contexts. This finding supports the implementation of evidence-based interventions and strategies to improve PL-related outcomes, particularly in the cognitive dimension. Moreover, the results from the PEKQ-S can guide professional development for PE teachers by highlighting areas where students lack conceptual understanding, prompting educators to adjust pedagogical approaches and enhance instructional quality in PE.

From a practical perspective, the PEKQ-S may serve as a useful foundational tool; however, its application in other Spanish-speaking contexts or educational stages would require appropriate cultural and curricular adaptation. Alignment with local physical education curricula and age-specific educational guidelines is essential to ensure the validity and relevance of the instrument beyond its original context. In terms of policy, as the first culturally adapted PL knowledge tool in Spain, the PEKQ-S can serve as a foundation for national and regional research studies and contribute data to international PL discussions. Its use in longitudinal or intervention studies will help track progress and the impact of PE reforms.

Finally, this study aligns with a recent article on the development of the first assessment model for PLs in Spain: the SPLA-C (32). In this Delphi study, national experts decided that this knowledge questionnaire should form part of this new assessment model for PLs in Spain. As part of the SPLA-C model, the PEKQ-S enables schools and policymakers to track students’ development in the cognitive domain of PL. This information can be used to design more holistic PE programs that address not only physical skills but also knowledge and understanding of movement, health, and physical activity.

Overall, the PEKQ-S was found to be a feasible, valid and reliable tool for assessing knowledge of PE content related to PL among schoolchildren (aged 6–12). The PEKQ-S clearly has a bifactor structure, demonstrating acceptable internal consistency and moderate to substantial test‒retest reliability for the majority of questions in a retest conducted within 15 days. It is also the first instrument to assess schoolchildren's knowledge of the cognitive domain in terms of PL and basic knowledge of the Spanish PE curriculum. The PEKQ-S is part of the first model of PL assessment in Spain: the SPLA-C.

## Data Availability

The raw data supporting the conclusions of this article will be made available by the authors, without undue reservation.
