# Supplementary material for: Development of the physical education knowledge questionnaire-Spain (PEKQ-S): feasibility, validity and reliability for Spanish children aged 6–12 years
Source: Front Sports Act Living. 2026 Jan 23;7:1737905. doi: 10.3389/fspor.2025.1737905 (PMC12876181; doi:10.3389/fspor.2025.1737905)
Supplement: Supplementary file 1 [file Supplementaryfile1.docx]

Supplementary Material 1

*A continuación, encontraréis un cuestionario para saber vuestro conocimiento sobre deporte y actividad física.*

*Me gustaría recordaros que es totalmente ANÓNIMO, es decir, nadie sabrá que las respuestas son tuyas.*

*No es un examen por lo que no hay respuestas buenas o malas, correctas o falsas. Responde todo lo que sepas, sin mirar las respuestas de tus compañeros. ☺*

- 1. ¿Piensas que es importante realizar actividad física o deporte habitualmente? ¿Por qué crees que es importante?
- ____________________________________________________________________________________________________________________________________________________________________________________________________________________________________________________________________________________________________________________________________
- _________________________________________________________________________________
- 2. Escribe 5 palabras que creas que es "ser saludable"
- __________________________________________________________________________________________________________________________________________________________________
- 3. Practicar actividad física o deporte es bueno porque… Escribe 3 motivos.
- ___________________________________________________________________________________________________________________________________________________________________________________________________________________________________________________
- _________________________________________________________________________________
- 4. ¿Cuánto tiempo crees que deben practicar actividad física o deporte los niños al día?
- *Con actividad física o deporte nos referimos a actividades que hacen que tu corazón lata más deprisa y que respires más rápido, como por ejemplo, caminar rápido o correr. *
- _________________________________________________________________________________
- 5. Escribe tres medidas (cosas, aspectos...) que debes tener en cuenta para realizar actividad física o deporte de forma más segura.
- ___________________________________________________________________________________________________________________________________________________________________________________________________________________________________________________
- 6. Después de practicar deporte, ¿qué haces para recuperarte y cuidar tu cuerpo?
- __________________________________________________________________________________________________________________________________________________________________
- _________________________________________________________________________________
- 7. ¿Por qué realizas deporte o actividad física?
- ___________________________________________________________________________________________________________________________________________________________________________________________________________________________________________________
- 8. ¿Qué sueles hacer en tu tiempo libre cuando no practicas deporte o actividad física? ¿Te gustan más esas actividades o hacer deporte?
- ___________________________________________________________________________________________________________________________________________________________________________________________________________________________________________________
- 9. Correr, lanzar, saltar, pillar y patear son habilidades que utilizamos cuando practicamos deporte o realizamos actividades físicas. ¿Por qué piensas que son importantes?
- ___________________________________________________________________________________________________________________________________________________________________________________________________________________________________________________
- 10. La fuerza, la velocidad, la resistencia y la flexibilidad son capacidades físicas. ¿Por qué piensas que son importantes cuando realizamos deporte o actividad física? ¿Puedes escribir un ejemplo?
- ___________________________________________________________________________________________________________________________________________________________________________________________________________________________________________________
- _________________________________________________________________________________
- 11. Escribe un ejemplo sobre cuando utilizas cada una de las capacidades físicas básicas:

a) un ejemplo de cuando utilizo la fuerza en el deporte, juego o actividad física

_________________________________________________________________________________

b) un ejemplo de cuando utilizo la velocidad en el deporte, juego o actividad física

_________________________________________________________________________________

c) un ejemplo de cuando utilizo la resistencia en el deporte, juego o actividad física

_________________________________________________________________________________

d) un ejemplo de cuando utilizo la flexibilidad en el deporte, juego o actividad física

_________________________________________________________________________________

12. ¿Eres capaz de controlar tus emociones? ¿Cómo lo haces? Por ejemplo, ¿si pierdes te enfadas con tus compañeros?

__________________________________________________________________________________________________________________________________________________________________

_________________________________________________________________________________

13. Escribe, dibuja o describe movimientos que puedan ayudarte a expresar tus emociones y pensamientos. Por ejemplo, cuando estas contento, enfadado, alegre, triste…etc.

14. Explica con tus palabras lo que creas que es la expresión corporal

__________________________________________________________________________________________________________________________________________________________________

_________________________________________________________________________________

_________________________________________________________________________________

15. ¿Podrías decirme tres diferencias entre un juego donde debes moverte y un juego donde no te mueves? Por ejemplo, las diferencias entre un juego de pillar y el "teléfono escacharrado".

___________________________________________________________________________________________________________________________________________________________________________________________________________________________________________________

_________________________________________________________________________________

16. ¿Por qué crees que es importante caminar o montar en bici en lugar de usar el coche? Escribe dos motivos por los que creas que es mejor desplazarte en bici o caminando en lugar de utilizar el coche

__________________________________________________________________________________________________________________________________________________________________

_________________________________________________________________________________

17. ¿Podrías decirme dos ejemplos de actividades físicas o deportes que se realicen en...?

- Agua: ______________________________________________________________________________
- Suelo:__
- ______________________________________________________________________________
- ¿y en el aire?:
- ______________________________________________________________________________

Supplementary Material 2

**Physical Education Knowledge Questionnaire for Spanish children (PEKQ-S)**

| Items | Cognitive interview |
| --- | --- |
| 1. ¿Crees que es importante realizar deporte o actividad física habitualmente? Marca la respuesta correcta.  a) Sí, es importante porque la actividad física/deporte es bueno para la salud.  b) Sí, es importante para tener un peso muy bajo.  c) No, no es importante. Simplemente es divertido.  d) No, no es importante. De hecho, hacer deporte es peligroso para la salud. | “Habitualmente” “normalmente” |
| 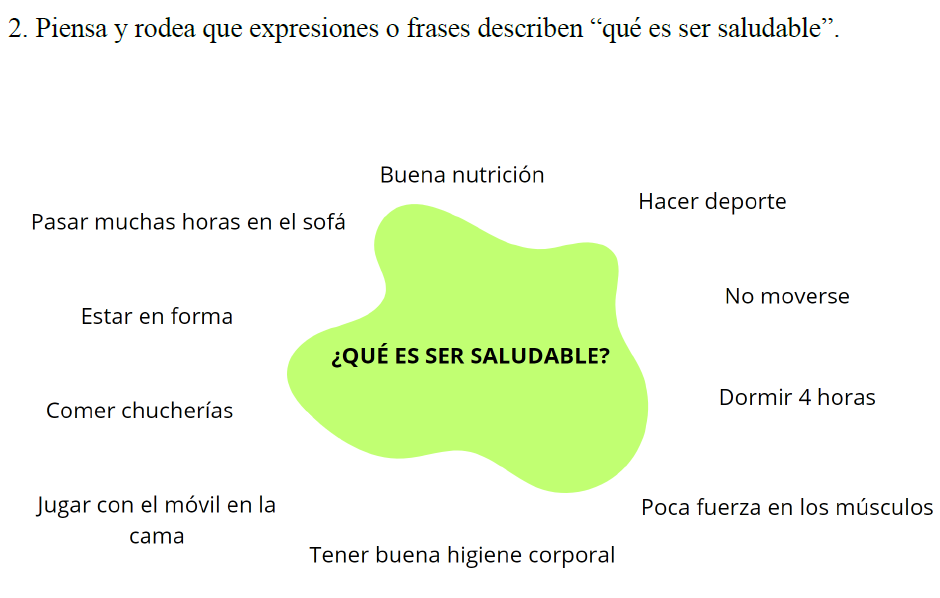 | “Nutrición” “alimentación”  “Higiene personal”  “estar limpio y aseado”  “Estar en forma” “estar fuerte o musculado” |
| 3. ¿Cuánto tiempo al día crees que deben practicar actividad física o deporte los niños? Señala la respuesta correcta.  a) máximo 20 minutos al día.  b) mínimo 1 hora al día.  c) 5 minutos al día.  d) 6 horas al día. |  |
| 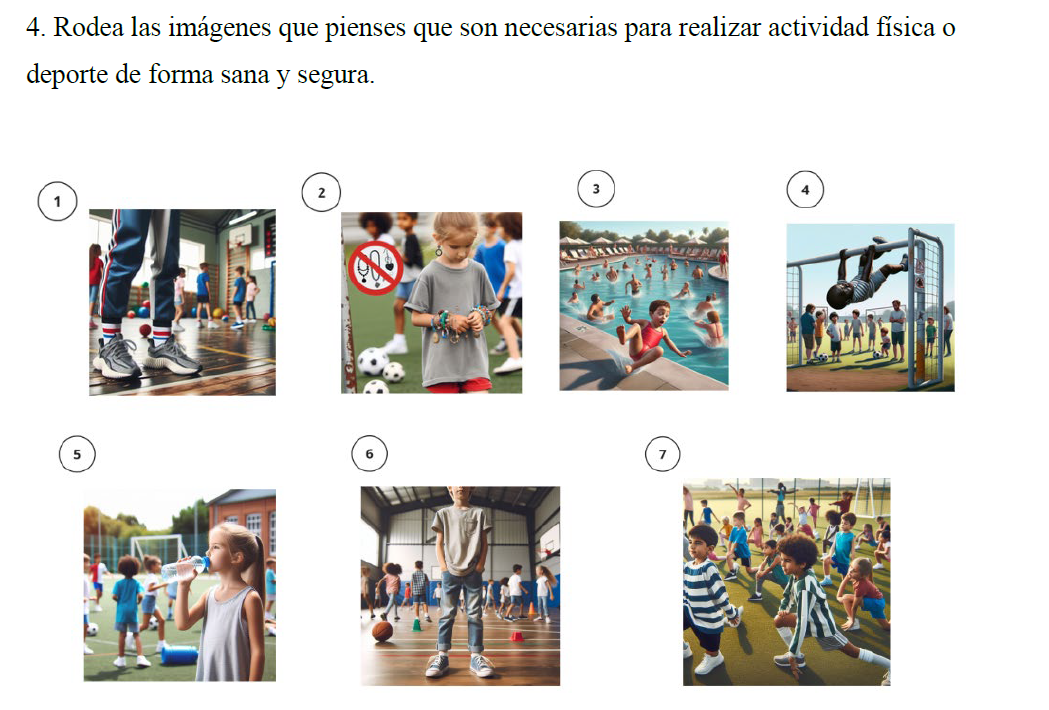 |  |
| 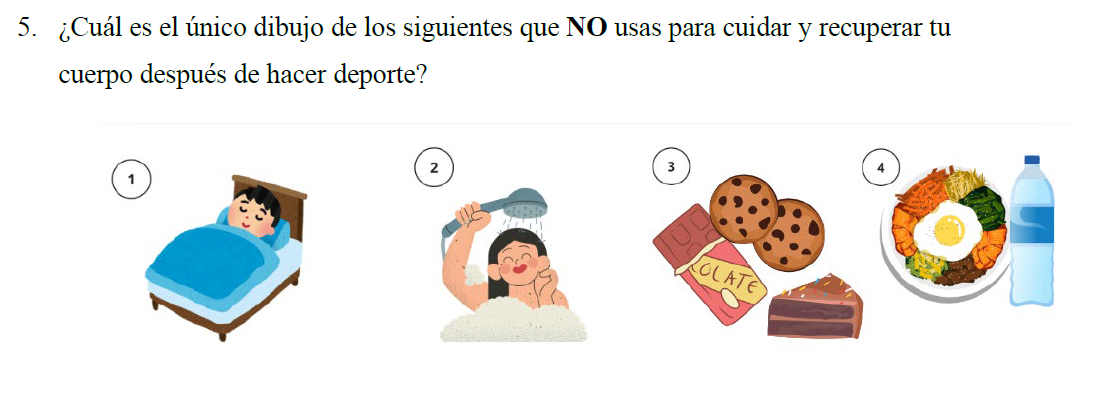 | “Descansar” “recuperar” |
| 6. No realizar actividad física o deporte de manera habitual puede… Señala la respuesta **correcta.**  a) mejorar el estado de ánimo.  b) ayudar a mantener el cuerpo en forma y fuerte.  c) ayudar a hacer nuevos amigos y trabajar en equipo con los demás.  d) aumentar el riesgo de tener enfermedades. | “Riesgo”  “peligro” |
| 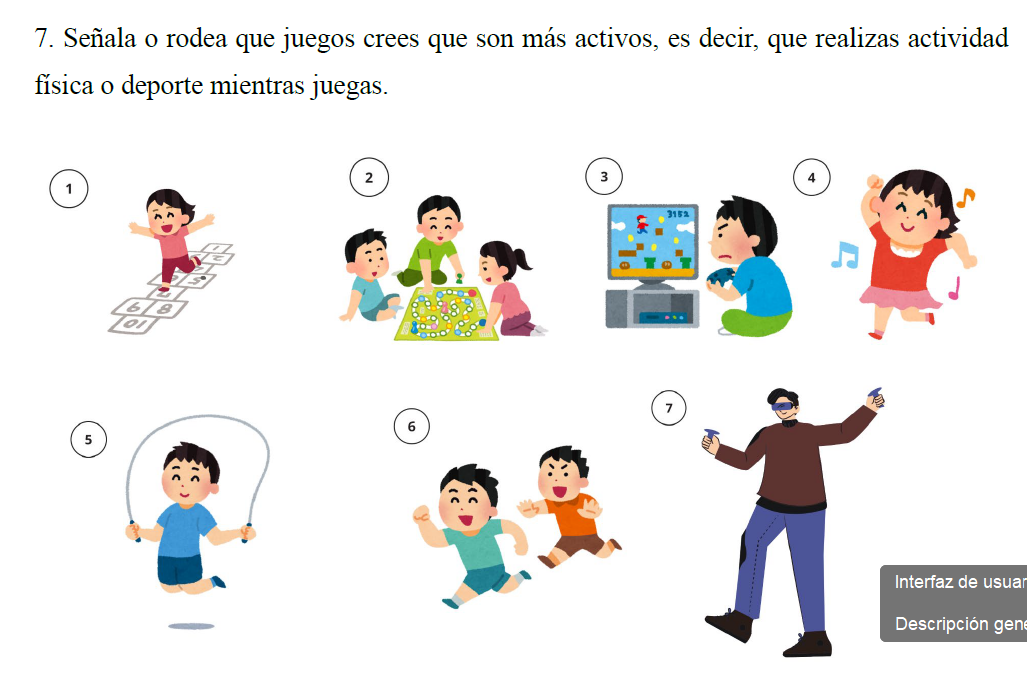 | “que realizas actividad física o deporte”  “que es moverse mientras juegas” |
| 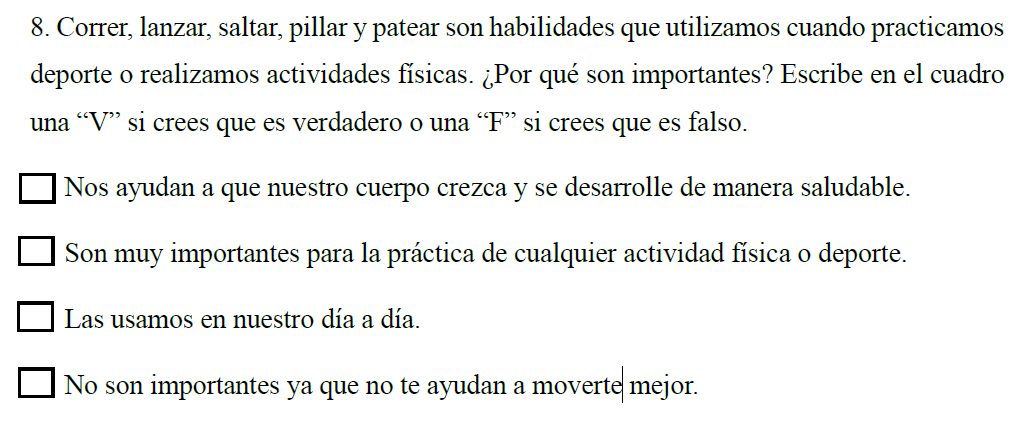 | “Desarrolle” “mejore” |
| 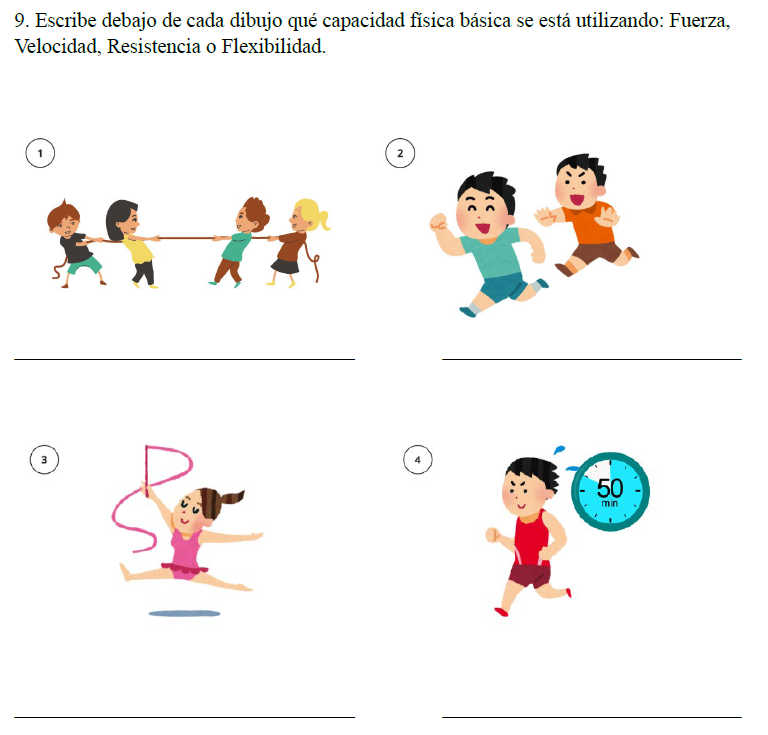 |  |
| 10. ¿Qué es la expresión corporal? Marca la respuesta correcta.  a) Consiste en sentarse adecuadamente.  b) Consiste en asearnos después de hacer deporte.  c) La manera en la que usamos nuestro cuerpo para comunicar algo (emociones, pensamientos…etc.) sin palabras.  d) La manera en la que escribimos con el lápiz. | “Consiste”  “es” |
| 11. ¿Crees que es importante caminar o montar en bici en tu día a día en lugar de usar el coche? (ejemplo: para ir al colegio, para quedar con un amigo o familiar que vive cerca de nuestra casa…etc.). Señala la respuesta **correcta.**  a) Sí es importante, porque es bueno para la salud y ayuda a mantenernos en forma.  b) No es importante, porque el coche es más rápido y saludable que montar en bici.  c) Sí es importante, es mejor ir en bici pero el problema es que contamina más que el coche.  d) No es importante, porque caminar y montar en bici es aburrido. | En forma por fuertes |
| 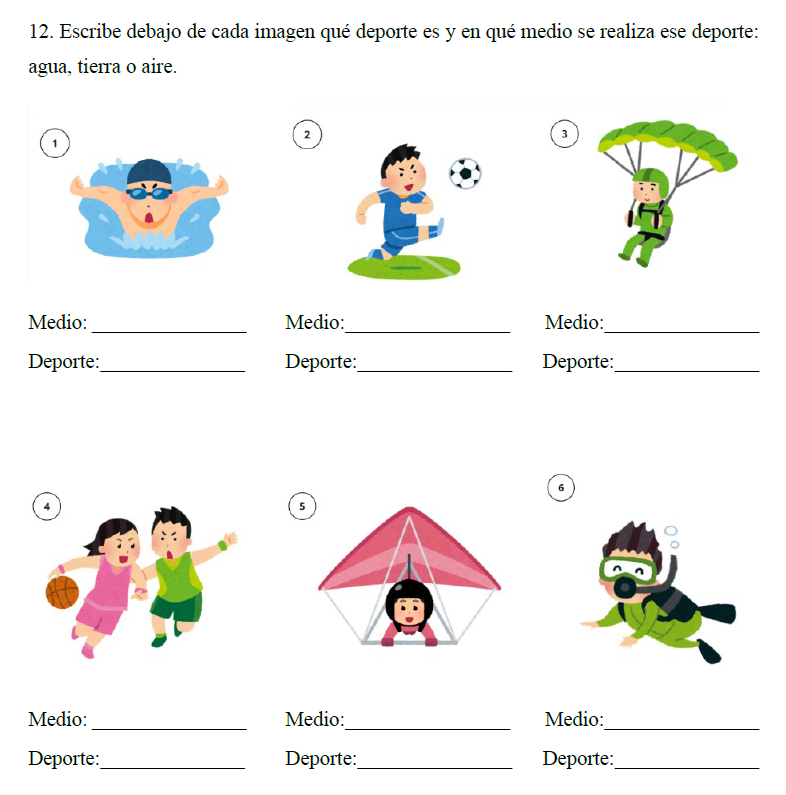 |  |

Supplementary Material 3

**Cuestionario sobre el conocimiento de los contenidos de Educación Física**

**Spanish Physical Literacy Assessment (SPLA)**

Fecha:

**Código:**

**En que curso estás actualmente**:
1º 2º 3º 4º 5º 6º *(por favor, rodea uno)*

**Eres**: chico chica *(por favor, rodea uno)*

**Qué mes es tu cumpleaños:** *(por favor, rodea uno****)***

En Feb Mar Abr May Jun Jul Ago Sept Oct Nov Dic

**Cuántos años tienes**:
6 7 8 9 10 11 12 13 14 *(por favor, rodea uno)*

En este proyecto, cuando hablamos de actividad física, nos referimos a cuando te mueves, juegas, haces ejercicio o deporte. Actividad física es cualquier actividad que haga que tu corazón lata más deprisa o que te deje sin aliento alguna vez.

¿Por qué te hacemos estas preguntas? Queremos saber qué piensan los niños como tú de la actividad física, el deporte y el ejercicio.

Por favor, recuerda:

☺ No hay respuestas correctas o incorrectas. Sólo queremos saber lo que piensas.

☺ Si no sabes una respuesta, escribe tu mejor suposición.

☺ No hay límite de tiempo, así que por favor tómate todo el tiempo que necesites.

1. ¿Crees que es importante realizar deporte o actividad física normalmente? Marca la respuesta correcta.

a) Sí, es importante porque la actividad física/deporte es bueno para la salud.

b) Sí, es importante para tener un peso muy bajo.

c) No, no es importante. Simplemente es divertido.

d) No, no es importante. De hecho, hacer deporte es peligroso para la salud.

2. Piensa y rodea que expresiones o frases describen “qué es ser saludable”.


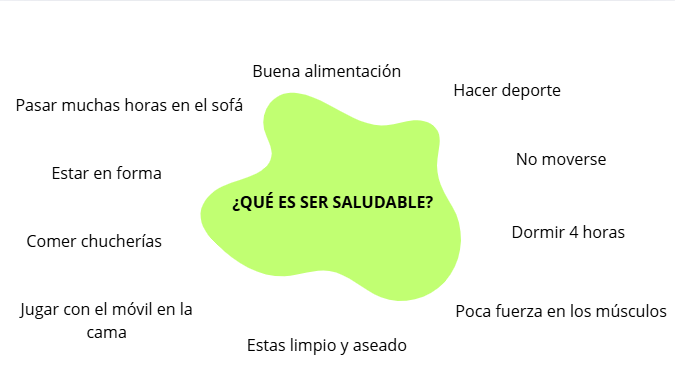


- 3. ¿Cuánto tiempo al día crees que deben practicar actividad física o deporte los niños? Señala la respuesta correcta.
- a) máximo 20 minutos al día.
- b) mínimo 1 hora al día.
- c) 5 minutos al día.
- d) 6 horas al día.
- 4. Rodea las imágenes que pienses que son necesarias para realizar actividad física o deporte de forma sana y segura.
-
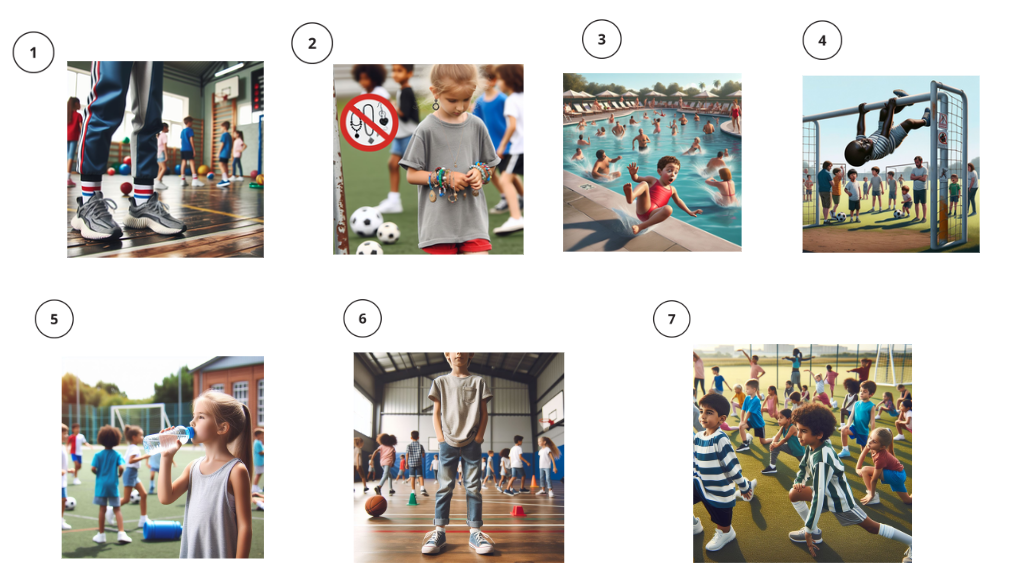


1. ¿Cuál es el único dibujo de los siguientes que **NO** usas para cuidar y descansar tu cuerpo después de hacer deporte?


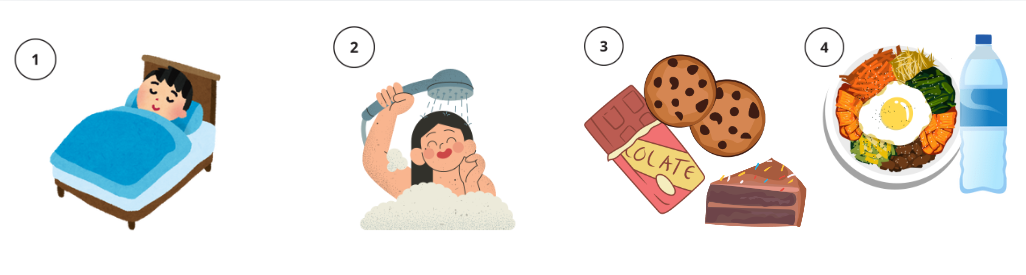


6. No realizar actividad física o deporte de manera habitual puede… Señala la respuesta **correcta.**

a) mejorar el estado de ánimo.

b) ayudar a mantener el cuerpo en forma y fuerte.

c) ayudar a hacer nuevos amigos y trabajar en equipo con los demás.

d) aumentar el peligro de tener enfermedades.

7. Señala o rodea que juegos crees que son más activos, es decir, que te mueves mientras juegas.


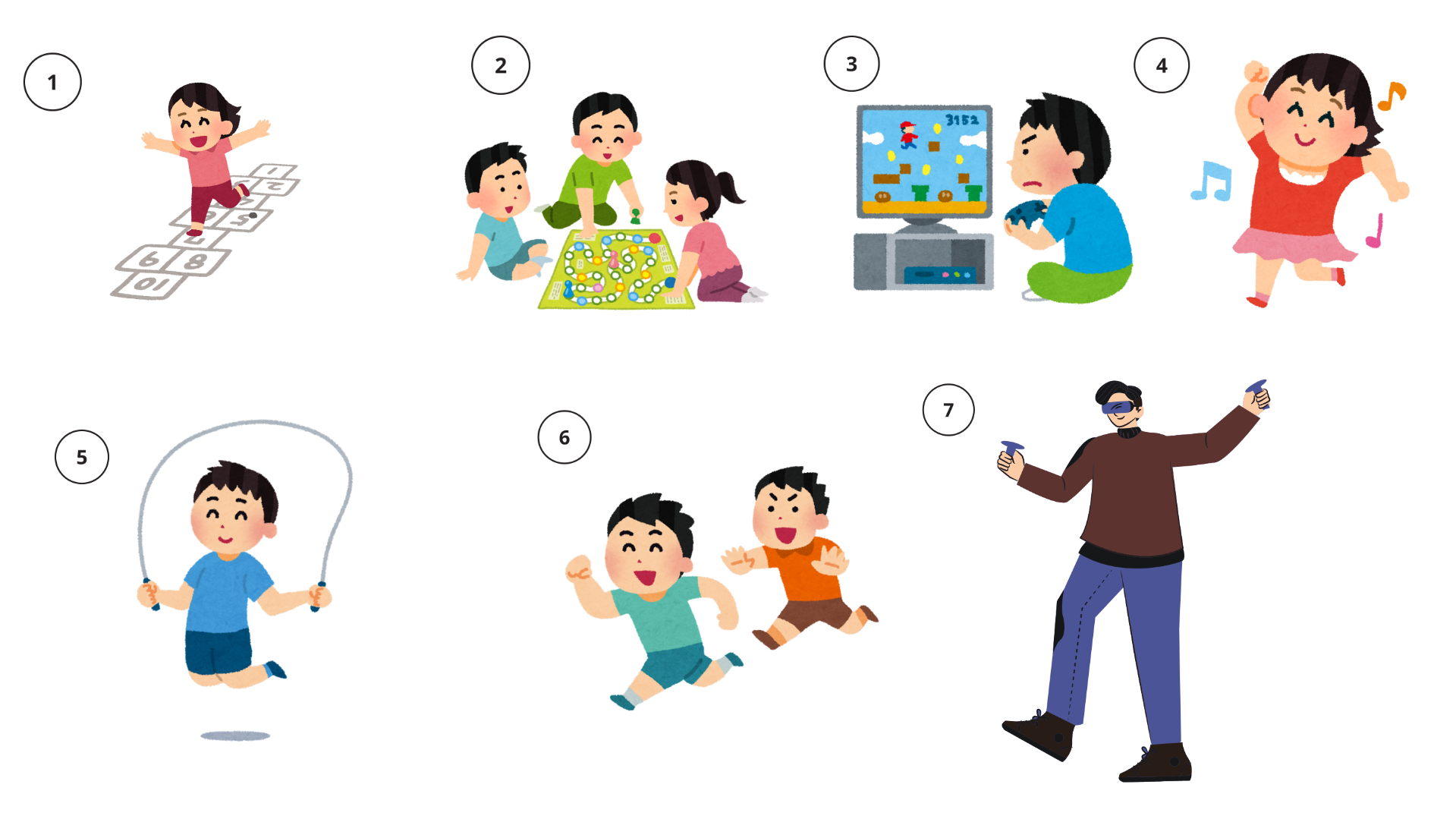


- 8. Correr, lanzar, saltar, pillar y patear son habilidades que utilizamos cuando practicamos deporte o realizamos actividades físicas. ¿Por qué son importantes? Escribe en el cuadro una “V” si crees que es verdadero o una “F” si crees que es falso.

Nos ayudan a que nuestro cuerpo crezca y se desarrolle de manera saludable.

Son muy importantes para la práctica de cualquier actividad física o deporte.

Las usamos en nuestro día a día.

No son importantes ya que no te ayudan a moverte mejor.

9. Escribe debajo de cada dibujo qué capacidad física básica se está utilizando: Fuerza, Velocidad, Resistencia o Flexibilidad.


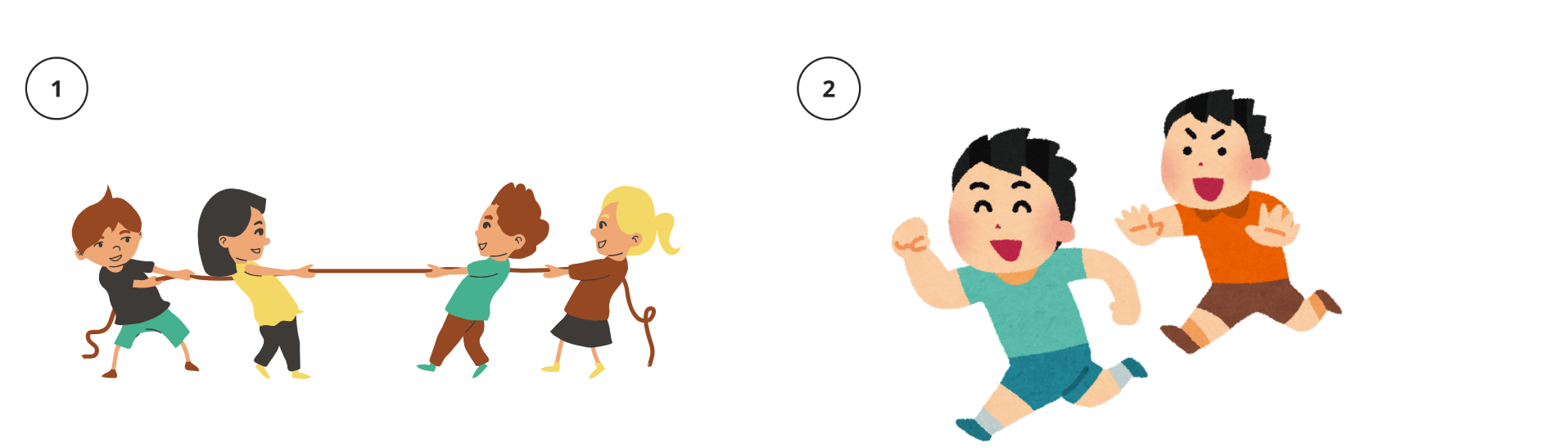


_________________________________ _____________________________


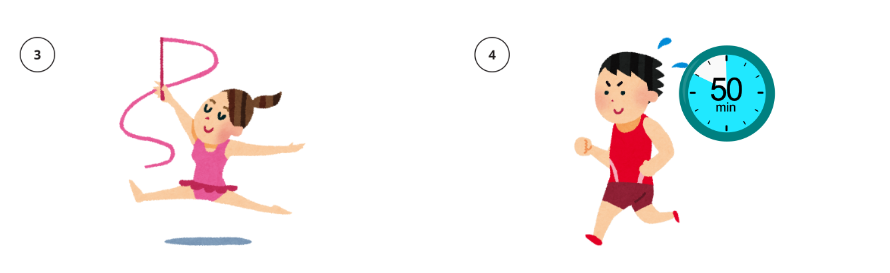


_________________________________ _____________________________

10. ¿Qué es la expresión corporal? Marca la respuesta correcta.

a) Es sentarse adecuadamente.

b) Es asearnos después de hacer deporte.

c) La manera en la que usamos nuestro cuerpo para comunicar algo (emociones, pensamientos…etc.) sin palabras.

d) La manera en la que escribimos con el lápiz.

11. ¿Crees que es importante caminar o montar en bici en tu día a día en lugar de usar el coche? (ejemplo: para ir al colegio, para visitar a un amigo o familiar que vive cerca de nuestra casa…etc.). Señala la respuesta **correcta.**

a) Sí es importante, porque es bueno para la salud y ayuda a mantenernos en buena forma.

b) No es importante, porque el coche es más rápido y saludable que montar en bici.

c) Sí es importante, es mejor ir en bici pero el problema es que contamina más que el coche.

d) No es importante, porque caminar y montar en bici es aburrido.

12. Escribe debajo de cada imagen qué deporte es y en qué lugar se realiza ese deporte: agua, tierra o aire.


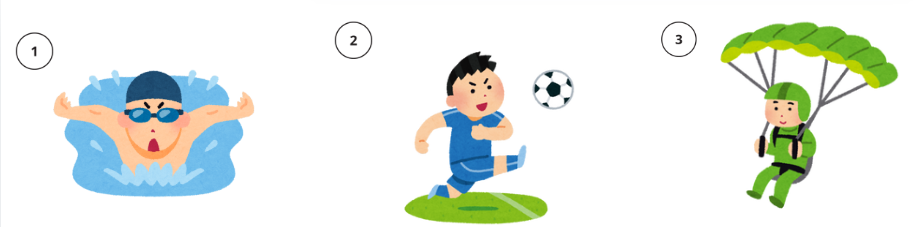


Medio: _______________ Medio:________________ Medio:_______________

Deporte:______________ Deporte:_______________ Deporte:______________


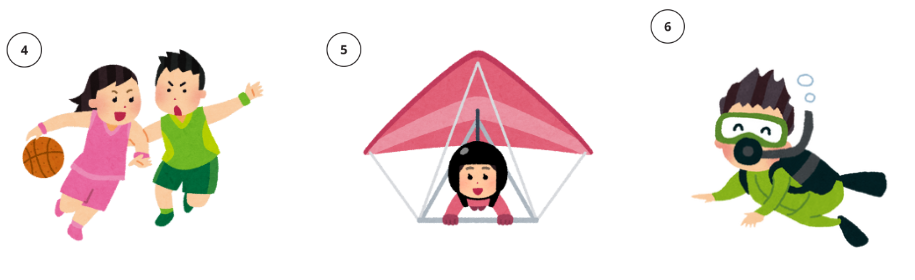


Medio: _______________ Medio:________________ Medio:_______________

Deporte:______________ Deporte:_______________ Deporte:______________

Supplementary Material 4

**Cuestionario sobre el conocimiento de los contenidos de Educación Física**

**Spanish Physical Literacy Assessment (SPLA)**

Fecha:

**Código:**

**En que curso estás actualmente**:
1º 2º 3º 4º 5º 6º *(por favor, rodea uno)*

**Eres**: chico chica *(por favor, rodea uno)*

**Qué mes es tu cumpleaños:** *(por favor, rodea uno****)***

En Feb Mar Abr May Jun Jul Ago Sept Oct Nov Dic

**Cuántos años tienes**:
6 7 8 9 10 11 12 13 14 *(por favor, rodea uno)*

En este proyecto, cuando hablamos de actividad física, nos referimos a cuando te mueves, juegas, haces ejercicio o deporte. Actividad física es cualquier actividad que haga que tu corazón lata más deprisa o que te deje sin aliento alguna vez.

¿Por qué te hacemos estas preguntas? Queremos saber qué piensan los niños como tú de la actividad física, el deporte y el ejercicio.

Por favor, recuerda:

☺ No hay respuestas correctas o incorrectas. Sólo queremos saber lo que piensas.

☺ Si no sabes una respuesta, escribe tu mejor suposición.

☺ No hay límite de tiempo, así que por favor tómate todo el tiempo que necesites.

1. ¿Crees que es importante realizar deporte o actividad física normalmente? Marca la respuesta correcta.

a) Sí, es importante porque la actividad física/deporte es bueno para la salud.

b) Sí, es importante para tener un peso muy bajo.

c) No, no es importante. Simplemente es divertido.

d) No, no es importante. De hecho, hacer deporte es peligroso para la salud.

2. Piensa y rodea que expresiones o frases describen “qué es ser saludable”.


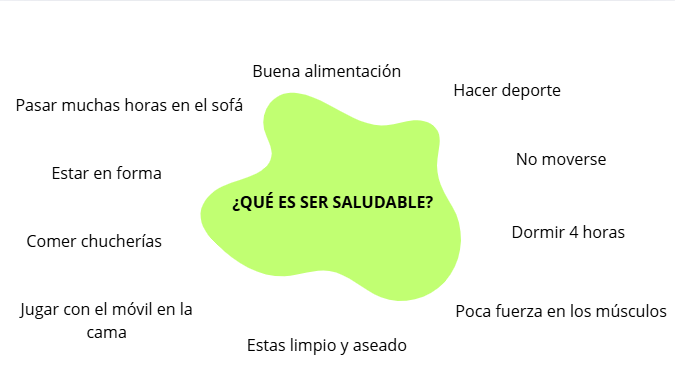


- 3. ¿Cuánto tiempo al día crees que deben practicar actividad física o deporte los niños? Señala la respuesta correcta.
- a) máximo 20 minutos al día.
- b) mínimo 1 hora al día.
- c) 5 minutos al día.
- d) 6 horas al día.
- 4. Rodea las imágenes que pienses que son necesarias para realizar actividad física o deporte de forma sana y segura.
-
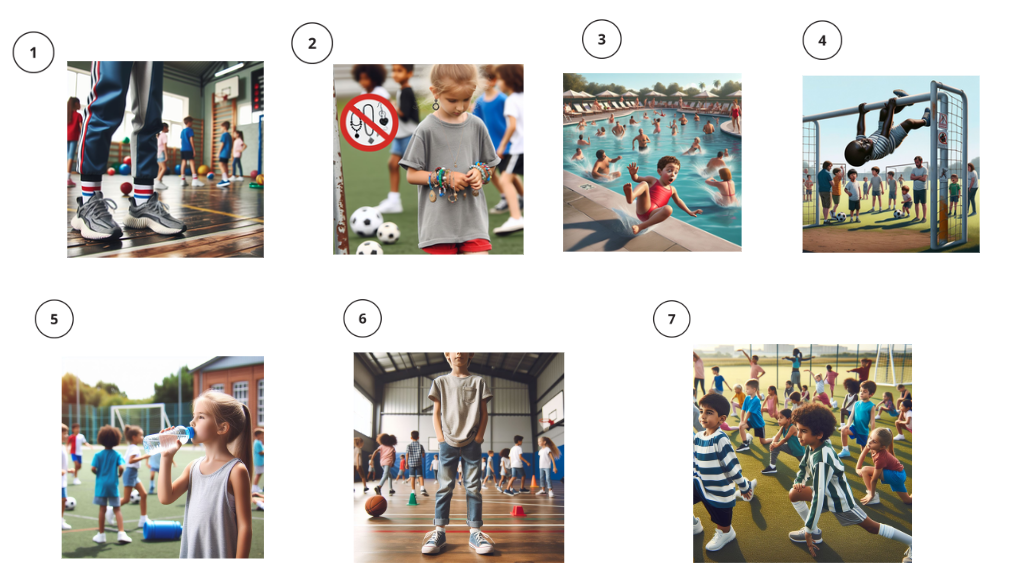


5. No realizar actividad física o deporte de manera habitual puede… Señala la respuesta **correcta.**

a) mejorar el estado de ánimo.

b) ayudar a mantener el cuerpo en forma y fuerte.

c) ayudar a hacer nuevos amigos y trabajar en equipo con los demás.

d) aumentar el peligro de tener enfermedades.

6. Señala o rodea que juegos crees que son más activos, es decir, que te mueves mientras juegas.


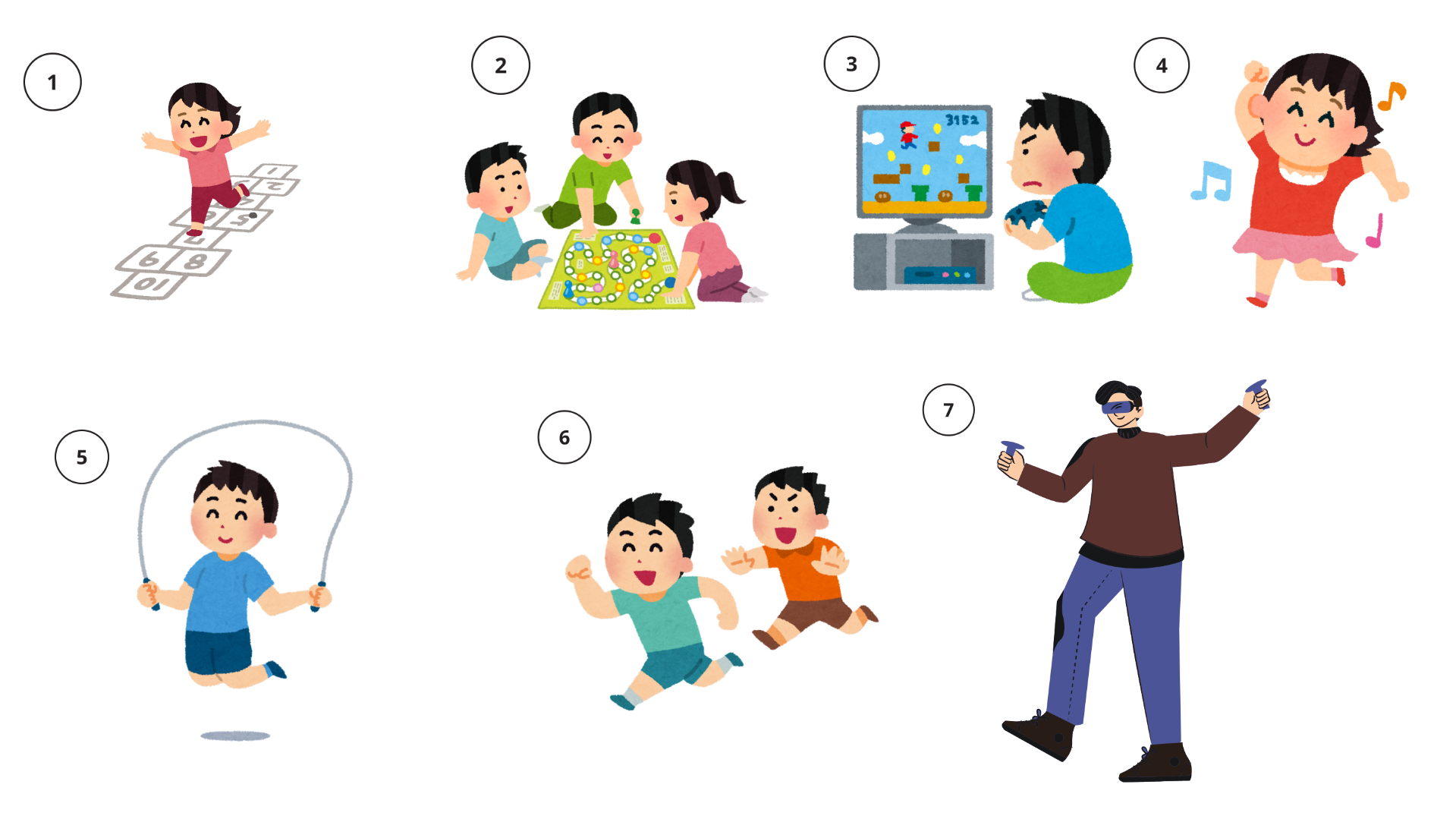
7. Escribe debajo de cada dibujo qué capacidad física básica se está utilizando: Fuerza, Velocidad, Resistencia o Flexibilidad.


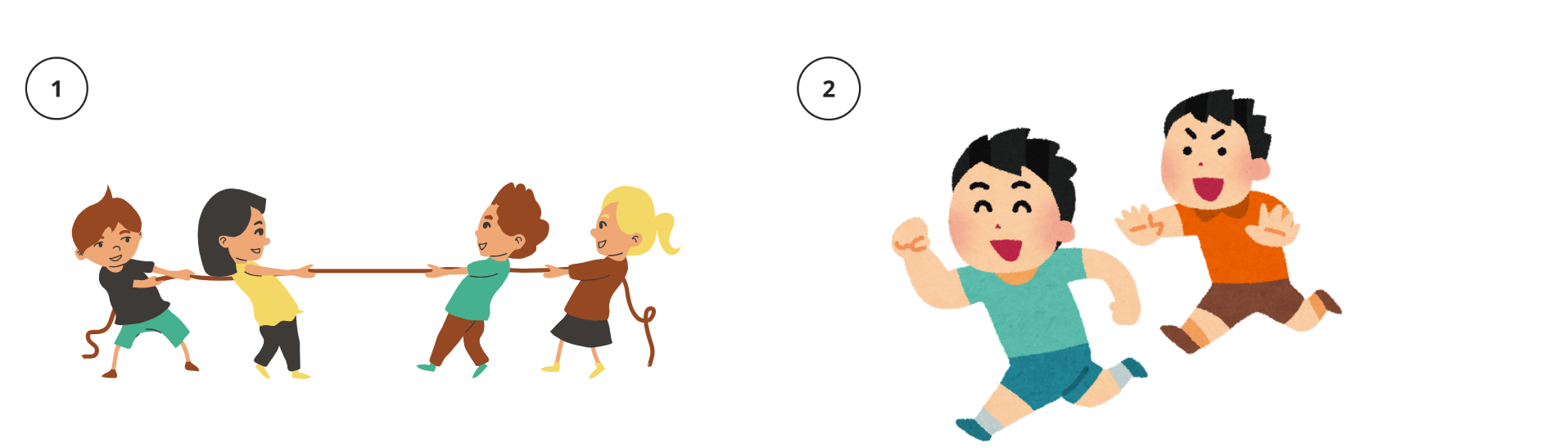


_________________________________ _____________________________


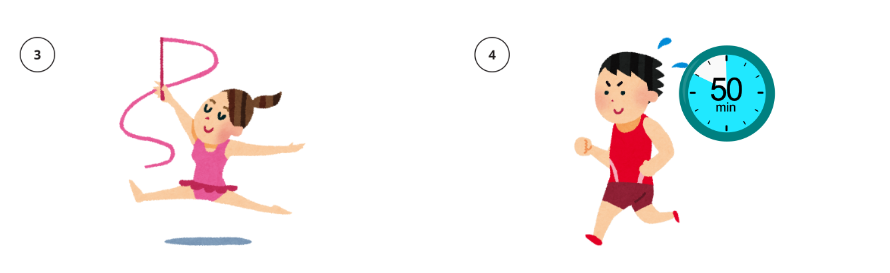


________________________________ _____________________________

8. ¿Qué es la expresión corporal? Marca la respuesta correcta.

a) Es sentarse adecuadamente.

b) Es asearnos después de hacer deporte.

c) La manera en la que usamos nuestro cuerpo para comunicar algo (emociones, pensamientos…etc.) sin palabras.

d) La manera en la que escribimos con el lápiz.

9. Escribe debajo de cada imagen qué deporte es y en qué medio se realiza ese deporte: agua, tierra o aire.


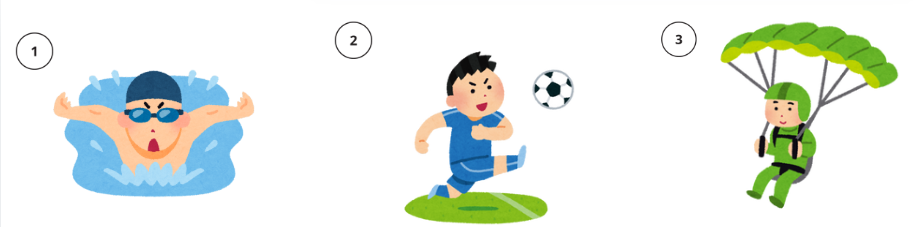


Medio: _______________ Medio:________________ Medio:_______________

Deporte:______________ Deporte:_______________ Deporte:______________


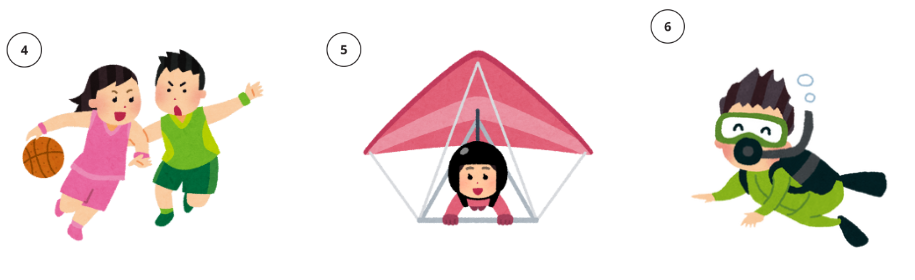


Medio: _______________ Medio:________________ Medio:_______________

Deporte:______________ Deporte:_______________ Deporte:______________
